# Comparative transcriptomics of closely related *Geobacter sulfurreducens* strains uncovers strain-level variations in extracellular electron transfer

**DOI:** 10.3389/fmicb.2026.1795027

**Published:** 2026-04-16

**Authors:** Mizuki Toda, Harima Takeuchi, Keisuke Tomita, Atsushi Kouzuma, Kengo Inoue, Kazuya Watanabe

**Affiliations:** 1 School of Life Sciences, Tokyo University of Pharmacy and Life Sciences, Hachioji, Tokyo, Japan; 2 Faculty of Agriculture, University of Miyazaki, Miyazaki, Japan

**Keywords:** bioelectrochemistry, conductive pili, cytochrome, electricity generation, nanowire, RNA sequencing

## Abstract

Members of *Geobacter sulfurreducens* are electroactive bacteria that transfer electrons to extracellular solid electron acceptors using extracellular electron transport (EET) pathways, thereby generating currents in bioelectrochemical systems (BESs). Studies have shown that different *G. sulfurreducens* strains generate different levels of current in BESs, while information is scarce as to molecular mechanisms behind strain-level variations in current generation. In order to gain insights into the strain-level variations, the present work comparatively analyzed *G. sulfurreducens* strains OSK2A and 60473 that are identified to be closely related in phylogenomic analyses. It is shown that 60473 generates larger current than OSK2A and forms thicker biofilms on electrodes. Comparative genomic analyses indicate that genes for catabolic and EET pathways in these strains are highly homologous (mostly over 99% identical in amino acid sequences). In contrast, comparative transcriptomics shows that expression levels of genes coding for extracellular proteins, including conductive pili and cytochrome nanowires, in the EET pathways are largely different between these strains, even though those for catabolic enzymes are relatively similar. These genes are expressed at substantially higher levels in strain 60473 than those in strain OSK2A. These results suggest that different levels of current generated by closely related *G. sulfurreducens* strains are attributable to differences in the expression of genes for extracellular proteins in the EET pathway.

## 1 Introduction

Members of the genus *Geobacter* and related genera are obligate anaerobes that respire using a variety of metals, including soluble ions and insoluble oxides (Lovley et al., 2011). For respiration using metal oxides that are deposited outside of cells, these bacteria exploit extracellular electron-transport (EET) pathways that span over inner and outer membranes for electron transfer between intracellular catabolic reactions and extracellular redox substances, including electrodes in bioelectrochemical systems (BESs) (White et al., 2016). These bacteria are therefore referred to as “electroactive bacteria (EAB),” and the discovery of EAB has prompted researchers to develop microbial electrochemical technologies (METs) that are expected to be applied to sustainable energy generation from biomass wastes and wastewater (Jadhav et al., 2022).

Currently, more than ten species are known within the genus *Geobacter*. In addition, recent work has proposed several *Geobacter*-related genera for species that were formerly classified within the genus *Geobacter*; these include *Geotalea daltonii* (Waite et al., 2020), *Geomonas bemidjensis* (Xu et al., 2019), *Pelotalea chapellei* (Xu et al., 2021) and *Geoanaerobacter pelophilus* (Xu et al., 2021). Studies have shown that, despite that all these bacteria possess substantial numbers (e.g., over 50) of genes for *c*-type cytochromes (cyts-c) (Soares et al., 2024), and some of them are considered to constitute EET pathways, their electrochemical activities are substantially different (Kato et al., 2013; Kato, 2017). For instance, Kato (2017) compared type strains of six *Geobacter*-related strains (*Geobacter sulfurreducens* PCA, *Geobacter metallireducens* GS-15, *Geotalea daltonii* FRC-32, *Geomonas bemidjensis* bem*, Pelotalea chapellei* 172, and *Geoanaerobacter pelophilus* Dfr2) for current generation in electrochemical cells, showing that strain PCA attains the highest current densities (per electrode area) among them at all potentials examined in the work (from –0.1 V to + 0.4 V vs. standard hydrogen electrode). It is therefore of interest to uncover how *G. sulfurreducens* is able to attain high current densities compared to other *Geobacter* strains. On the other hand, comparative genomic analyses of six *Geobacter*-related strains, including *G. sulfurreducens*, suggest that they have common pathways for acetate oxidation and energy conservation, while the paths of electrons across outer membrane would be variable (Butler et al., 2010).

Studies have also shown that different strains of EAB affiliated with *G. sulfurreducens* exhibit different levels of electrochemical activities (Yi et al., 2009; Fujikawa et al., 2021; Harada et al., 2025a). For instance, currents generated by strains PCA, KN400, YM18 and YM35 (all affiliated with *G. sulfurreducens*) were compared under same experimental conditions, demonstrating that current densities attained by these strains are different (Fujikawa et al., 2021). It has also been shown that strains PCA, YM18, and 60473 develop structurally different biofilms on electrodes, resulting in different levels of electrochemical activities (Harada et al., 2025a). On the other hand, comparative genomics of *G. sulfurreducens* strains DL-1 and KN400 has shown that the improvement in EET in KN400 does not appear to be due to novel gene acquisition, but rather to changes in the common metabolic network (Butler et al., 2012). Despite these studies, however, molecular mechanisms behind the strain-level variation in electrochemical activities of *G. sulfurreducens* still remain not to be sufficiently understood.

In the present work, after phylogenomic analyses were conducted to identify relatedness of seven *G. sulfurreducens* strains whose complete genomes are deposited in the databases (strains PCA, KN400, YM18, YM35, PL, OSK2A, and 60473), two closely related strains (OSK2A and 60473) were selected for comparative physiological, genomic and transcriptomic analyses. We aim at gaining insights into molecular mechanisms behind strain-level variations in electrochemical activities of *G. sulfurreducens* EAB.

## 2 Materials and methods

### 2.1 Bacterial strains and culture conditions

*G. sulfurreducens* strain 60473 was isolated from lakeshore mud (Harada et al., 2025a) and maintained in our laboratory. Its complete genome sequence has been reported (Harada et al., 2024). *G. sulfurreducens* strain OSK2A (Viulu et al., 2013) was purchased from Japan Collection of Microorganisms. Cells of these strains were maintained on agar plates containing DSM826 medium in anaerobic bags (AnaeroPack; Mitsubishi Gas Chemical Company, Tokyo, Japan). These strains were routinely cultivated in DSM826 medium, in which acetate (10 mM) and fumarate (50 mM) serve as the electron donor and acceptor, respectively. They were grown at 30°C under strict anaerobic conditions as described elsewhere (Kato et al., 2010). Growth in DSM826 medium was monitored by measuring the optical density at 600 nm (OD_600_) using a mini photo 518R photometer (TAITEC Corp., Tokyo, Japan). Growth on ferric iron as an electron acceptor was examined by measuring ferrous iron as described elsewhere (Jiménez Otero et al., 2018). Liquid cultures were preserved at –80°C in the presence of 20% dimethyl sulfoxide.

### 2.2 Phylogenetic and phylogenomic analyses

Complete genome sequences are available for 7 strains of *G. sulfurreducens*, including strains PCA, KN400, YM18, YM35, PL, OSK2A, and 60473, and these sequences were retrieved from the NCBI Genome database.^1^ Genomic relatedness of these strains was examined using Genome-to-Genome Distance Calculator (GGDC) version 3.0 (Meier-Kolthoff et al., 2022) and Average Nucleotide Identity (ANI) Calculator (Yoon et al., 2017). A phylogenomic tree based on the complete genome sequences was constructed using DiGAlign (Nishimura et al., 2024). Phylogenetic relationships among the 7 strains were analyzed using 16S rRNA gene and *gyrB* sequences, and the alignment of these sequences and construction of phylogenetic trees were conducted using MEGA11 (Tamura et al., 2021). Sequences of these genes were retrieved from their genome sequences.

### 2.3 Analyses of electrochemical activities

Single-chamber three-electrode electrochemical cells (ECs, 95 mL in capacity) were used to evaluate current generation in pure cultures. An EC was equipped with a graphite-felt (GF) working electrode (WE; 1 cm^2^ in projected area, 3 mm in thickness; GF-20-5F, Nippon carbon, Tokyo, Japan), an Ag/AgCl reference electrode [RE; + 0.2 V vs. standard hydrogen electrode (SHE); RE-T7A, EC Frontier, Kyoto, Japan], and a counter electrode (CE; platinum mesh; 2 cm^2^; Nilaco, Tokyo, Japan). The EC was filled with 65 mL of fumarate-free DSM826 medium, and, after purged with high-purity nitrogen gas (99.999%) and supplemented with L-cysteine (5 mM), it was inoculated with a bacterial culture pre-grown in DSM826 medium. The initial OD_600_ was 0.02. An EC was connected to a potentiostat (VMP3; BioLogic, Seyssinet-Pariset, France), and, after the WE potential was poised at 0 V (vs. SHE), current (*I*, mA) was measured at 30°C and recorded. A current density (*J*, mA cm^–2^) was calculated based on the projected area of WE, and the highest *J* recorded during the operation was termed *J*_*max*_ (mA cm^–2^).

After the operation (approx. 5 days), WE was taken from an EC and soaked in fumarate-free DSM826 medium containing 4,6-diamidino-2-phenylindole (DAPI, 0.1 μg mL^–1^) for 10 min. Subsequently, WE was transferred to fumarate-free DSM826 medium and completely soaked in it for observing fully hydrated biofilms attaching onto WE under confocal laser scanning microscopy (CLSM) using an FV1200/BX61WI microscope system (Olympus, Tokyo, Japan) according to manufacturer’s manuals. An amount of biofilm attaching onto WE was determined by a protein assay as described elsewhere (Harada et al., 2025a).

### 2.4 Genomic analyses

DigAlign was used for constructing a synteny map (Nishimura et al., 2024). Genes for catabolic and EET pathways in the genomes of strains 60473 (AP028967.1) and OSK2A (AP028061.1) were identified based on the annotation in the NCBI database and BLAST search (Altschul et al., 1990) using annotated-gene sequences in the genome of strain PCA. Homology search was conducted using the BLAST program. Alignment of nucleotide sequences in intergenic regions was conducted by the ClustalW program in the GenomeNet website.^2^

### 2.5 Transcriptomic analyses

RNA was extracted from cells of strains 60473 and OSK2A grown under three different conditions; namely, planktonic cells grown using fumarate as the electron acceptor (PF), biofilm cells grown on fumarate and attaching onto GF (BF), and biofilm cells grown using the GF electrode as the electron acceptor in current-generating EC (BE). Triplicate cultures were prepared for each condition.

PF cells were grown in ECs (without the electrodes) containing DSM826 medium for 15 h. Planktonic cells were harvested by centrifugation at 10,000 × *g* for 5 min, and the pellet was immediately suspended in TRIzol reagent (Thermo Fisher Scientific, Waltham, MA). BF cells were grown in ECs that were equipped with GFs (as a support medium but not as an electrode) and filled with DSM826 medium. GFs covered with biofilms were taken out from ECs 15 h after commencing the cultivation and immediately soaked in TRIzol reagent. BE cells were grown on GF WEs (poised at 0 V vs. SHE) during current generation as described above. GF WEs covered with biofilms were taken out from ECs 15 h after commencing the cultivation and immediately soaked in TRIzol reagent. RNA was extracted and purified from cells in TRIzol reagent using RNeasy Mini Kit and RNase-Free DNase Set (Qiagen, Valencia, CA) according to manufacturer’s instructions. The quantity and quality of the purified RNA was checked using a NanoDrop ND-1000 spectrophotometer (Thermo Fisher Scientific) and an Agilent 2100 bioanalyzer with RNA 6000 Pico reagents and RNA Pico Chips (Agilent Technologies, Santa Clara, CA) according to manufacturer’s instructions.

RNA samples were subjected to rRNA removal using Ribo-Zero Plus rRNA depletion kit (Illumina, San Diego, CA), and cDNA libraries were prepared using NEBNext Ultra II Directional RNA Library Prep kit (New England Biolabs Inc., Ipswich, MA). The libraries were sequenced using the Illumina NovaSeq X Plus platform with a read length of 150 bp (paired ends). Approximately 1 GB of total reads were obtained for each library to attain the sufficient resolution of the mRNA profiles. The reads were trimmed, mapped on to the genome of strain 60473 or OSK2A and counted using CLC Genomics Workbench (version 8.5, Qiagen). A count for each gene was used to estimate a Reads Per Kilobase of exon per Million mapped reads (RPKM) value that represents an expression level of the gene. Highly expressed genes are defined as those whose RPKM values are larger than 1,000, since this criterion selects nearly 100 genes under each condition.

Differential gene expression (DGE) was also analyzed using an RNA-seq analytical tool in the CLC Genomics Workbench. A fold change (FC) denotes DGE in one strain between two culture conditions. Genes with adjusted *p*-value < 0.05 and log_2_FC below −1.0 or above + 1.0 were defined as differentially expressed (significantly up- or down-regulated, respectively). A fold difference (FD) denotes DGE of orthologous genes in strains 60473 and OSK2A under a same growth condition (RPKM in 60473 divided by RPKM in OSK2A). Genes with adjusted *p*-value < 0.05 and log_2_FD below −1.0 or above + 1.0 were defined as significantly differentially expressed.

Data were statistically analyzed by the *t-*test using the Welch’s method. A pair of data are determined to be significantly different, when a *p-*value was < 0.05. The raw RNA sequencing reads are available in the DDBJ Sequence Read Archive under accession numbers DRR900831-DRR900848.

## 3 Results and discussion

### 3.1 Phylogenetic and phylogenomic relatedness of *G. sulfurreducens* strains

**FIGURE 1:**
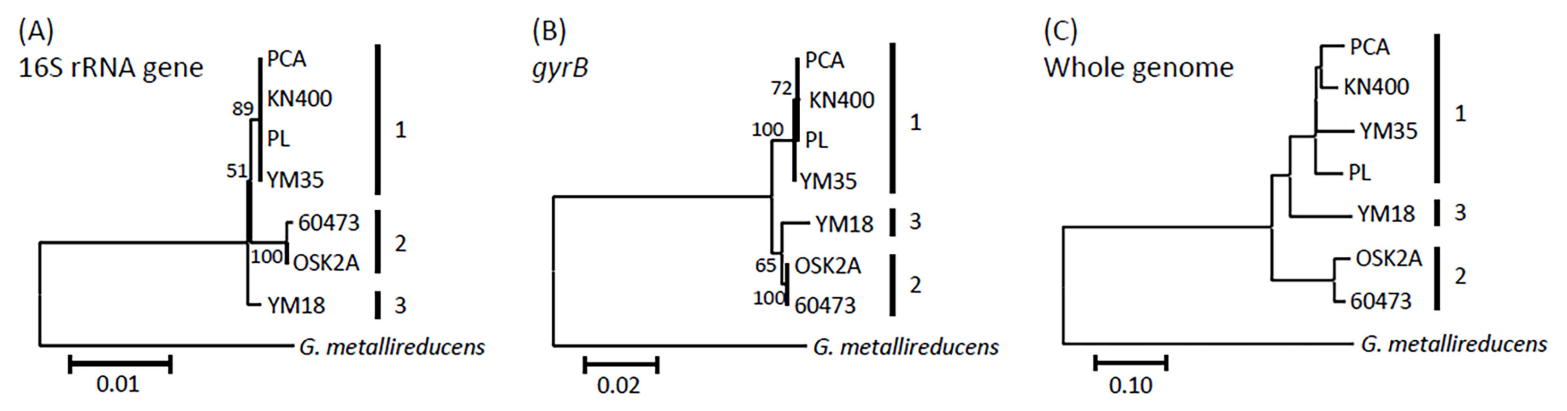
Phylogenetic and phylogenomic relationships among *G. sulfurreducens* strains whose complete genome sequences are deposited in the databases. **(A)** Phylogenetic relationships based on 16S rRNA gene sequences. **(B)** Phylogenetic relationships based on *gyrB* gene sequences. **(C)** Phylogenomic relationships based on whole genome sequences. Numbers at branch nodes are bootstrap values (100 trials). *G. metallireducens* serves as the outgroup. Scale bars are presented below the trees.

Genomic relatedness of seven *G. sulfurreducens* strains whose complete genomes are deposited in the NCBI database was analyzed. Even though these strains were isolated from distant regions across different countries, general genomic features, including genome sizes and G + C contents are similar (Supplementary Table 1). Phylogenetic relationships were analyzed based on 16S rRNA gene and *gyrB* sequences (panels A and B in Figure 1, respectively). The *gyrB* genes were used for the analysis, since this gene has been reported useful for dissecting relationships among closely related strains (Watanabe et al., 2001). The analyses revealed that these strains are divided into three sub-species groups (groups 1, 2, and 3). This idea is also supported by genomic analyses, including the phylogenomic analysis (Figure 1C) and the GGDC and ANI analyses (Supplementary Table 2). It is shown that strains PCA, KN400, PL, and YM35 are closely related, despite that they were isolated from various locations across the world (Supplementary Table 1). Strains 60473 and OSK2A are also closely related, and these were isolated from lake sediment (mud) in Japan (these lakes are ∼200 km apart) (Viulu et al., 2013; Harada et al., 2025a). Unlike other *G. sulfurreducens* strains, these two strains are known to be able to utilize ethanol as an electron donor (Viulu et al., 2013; Harada et al., 2025b). Previous studies have reported that these strains are active EAB (Viulu et al., 2013; Harada et al., 2025a), while their electrochemical activities have not yet been compared in same experimental setups.

### 3.2 Electrochemical activities of strains 60473 and OSK2A

**FIGURE 2:**
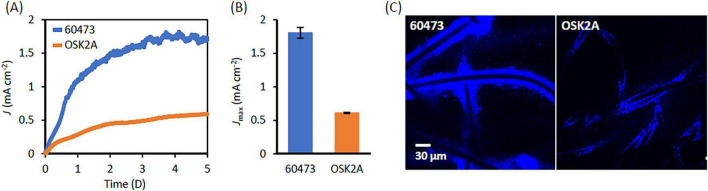
Current generation by *G. sulfurreducens* strains 60473 and OSK2A. **(A)** Representative time courses of current generation in fumarate-free DSM826 medium at WE potential of −0.2 V vs. Ag/AgCl RE. **(B)** Comparison in *J*_*max*_ values. An error bar represents SD (*n* = 3). **(C)** CLSM images for DAPI-strained biofilms formed on graphite fibers in WEs after current generation.

Electrochemical activities of strains 60473 and OSK2A were compared using the same EC system, in which acetate served as the electron donor (Figure 2). It is shown that their electrochemical activities are substantially different (Figure 2A); namely, 60473 generates more than 3 times the current compared to OSK2A (Figure 2B). It is interesting that even the most closely related strains exhibit markedly different electrochemical activities. On the other hand, growth tests show that strain OSK2A grows slightly faster than strain 60473 in liquid medium containing acetate as the electron donor and fumarate as the electron acceptor (Supplementary Figure 1A). In addition, no significant difference in the growth on ferric iron as an electron acceptor was observed between the two strains (Supplementary Figure 1B).

A previous study has shown that different *G. sulfurreducens* strains form different shapes of biofilms on electrodes (Harada et al., 2025a). The present work therefore compared biofilms formed on WEs by strains 60473 and OSK2A after current generation, and CLSM images show that 60473 forms thicker biofilms than OSK2A (Figure 2C). In addition, amounts of proteins attaching onto WEs were measured as indices for biofilm quantity, showing that more proteins were measured in 60473-inoculated ECs than those in OSK2A-inoculated ECs (Supplementary Figure 2). More biofilms were also observed by CLSM on the WE materials in 60473-inoculated ECs under the BF condition than those in OSK2A-inoculated ECs (data not shown). It is likely that the superior ability of strain 60473 to form biofilms on GF WEs facilitates current generation by this strain.

### 3.3 Comparative genomics of strains 60473 and OSK2A

The above experiments demonstrate that current generation and biofilm formation are largely different even between *G. sulfurreducens* strains that are closely related in phylogenetic and phylogenomic analyses. A similar phenomenon has also been found in previous studies that compared strains DL1 and KN400 (Yi et al., 2009). Even though these strains are closely related, their electrochemical activity and biofilm formation are largely different (Yi et al., 2009). Comparative genomics of strains PCA and KN400 have shown that the EET pathways of these strains, including conductive pili, are unchanged, and it has been concluded that changes in carbon flux and ATP metabolism may alter the electrical connection between the cell and electrode (Butler et al., 2012).

In the present study, comparative genomics of strains 60473 and OSK2A was conducted with a focus on their catabolic and EET pathways. As shown in Supplementary Table 1, the genome of strain OSK2A is approx. 200 kb longer than that of strain 60473, and this difference is ascribable to one insertion sequence in the OSK2A genome (Supplementary Figure 3). Global synteny analysis shows that gene orders in their genomes are generally in good agreement, while there is an inversion of a large genomic region of approximately 1 Mb (Supplementary Figure 3). It is also seen in this figure that most of corresponding genes in their genomes are highly homologous (over 95% identical in nucleotide sequence).

**FIGURE 3:**
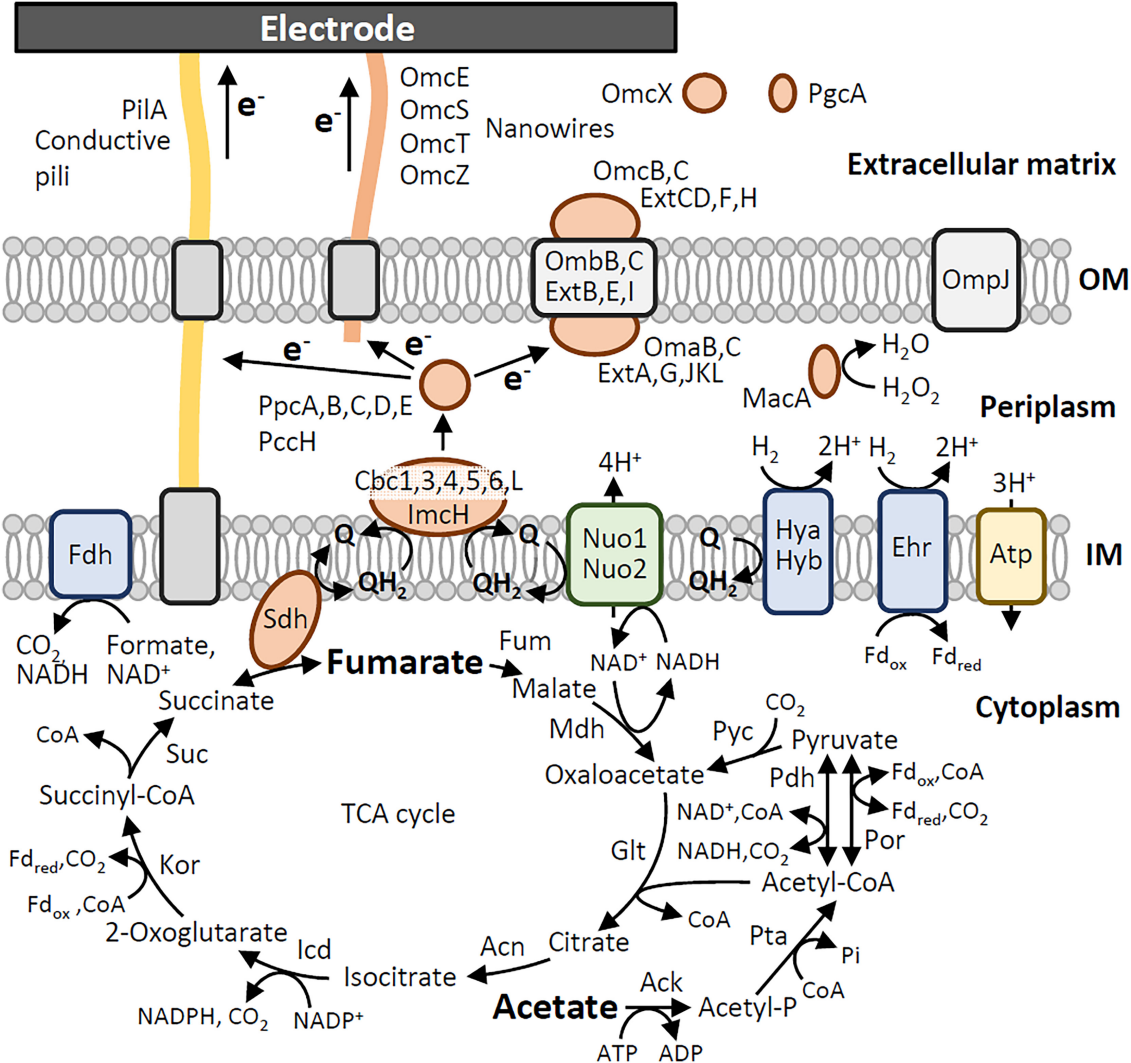
Catabolic and EET pathways for current generation from acetate in *G. sulfurreducens* strains 60473 and OSK2A reconstructed from the genomic data. IM, inner membrane; OM, outer membrane.

Based on results of Blast search using annotated genes in strain PCA and knowledge from molecular studies on strain PCA (Aklujkar et al., 2013; Ueki, 2021; Gralnick and Bond, 2023; Zakizadeh Tabari and Hochbaum, 2025), orthologous genes encoding proteins that would be involved in current generation from acetate in strains 60473 and OSK2A are listed (Supplementary Table 3), and catabolic and EET pathways in these strains are reconstructed in Figure 3. In these pathways, all genes orthologous to those of strain PCA are found in the genomes of strains 60473 and OSK2A; these include genes for 2 NADH:quinone oxidoreductases (Nuo1 and Nuo2), 7 inner membrane (IM) quinone:cyt-c oxidoreductases (Cbc1, Cbc3, Cbc4, Cbc5, Cbc6, CbcL, and ImcH), 6 periplasmic cyts-c (PpcA, PpcB, PpcC, PpcD, PpcE, and PccH), 5 outer-membrane (OM) porin/cyt-c complexes (PCCs; OmaB/OmbB/OmcB, OmaC/OmbC/OmcC, ExtABCD, ExtEFG, and ExtHIJKL), a conductive pilus (PilA) and 4 extracellular cyt-c nanowires (OmcE, OmcS, OmcT, and OmcZ). It is shown that, similar to strain PCA, the EET pathways in strains 60473 and OSK2A are highly redundant. Previous studies have shown that gene knockout does not necessarily lead to phenotype changes due to the presence of functionally complementary genes (Jiménez Otero et al., 2018; Choi et al., 2022). It is likely that similar results would be obtained in gene-knockout examinations of strains 60473 and OSK2A for dissecting their EET pathways.

Homology search shows that catabolic and EET genes in strains 60473 and OSK2A are highly homologous (mostly over 99% identical in nucleotide and amino-acid sequences) (Supplementary Table 3). Accordingly, it is concluded that the differences in current generation between these strains cannot be explained based on the comparative genomic analyses.

Biofilms formed by *G. sulfurreducens* are “electroactive biofilms (EABF)” that are prerequisite for current generation (Zhao et al., 2021). It has been discovered that EABF are composed of a variety of secreted materials, including polysaccharides, proteins and DNAs (You et al., 2023), and studies have attempted to identify genes necessary for EABF formation (such as Rollefson et al., 2009). Despite these efforts, however, since the formation process, composition and regulatory mechanisms of EABF are very complex, only a portion of genes involved in EABF formation appear to be understood so far. Comprehensive analyses of genes involved in EABF formation by strains 60473 and OSK2A are therefore difficult, while some genes known to be essential for EABF formation in strain PCA, such as GSU1501 (Rollefson et al., 2009) and GSU1658 (Hallberg et al., 2016), are found to be conserved in these strains.

### 3.4 Transcriptomics under three growth conditions

We hypothesized that, although there are no substantial differences between strains 60473 and OSK2A in genes encoding the catabolic and EET pathways (Supplementary Table 3), expression levels of these genes in the two strains would be different, resulting in the difference in current generation (Figure 2). In order to address this idea, the present study conducted comparative transcriptomics of strains 60473 and OSK2A, and RNA-seq was performed under three growth conditions, namely, PF, BF, and BE, as described in the methods section. It is expected that comparisons in transcription levels of genes between the PF and BF conditions would facilitate the detection of genes that are up- or down-regulated in association with biofilm formation, while comparisons between the BF and BE conditions would facilitate the detection of genes that are up- or down-regulated in association with current generation. A summary of RNA-seq results is presented in Supplementary Table 4, in which numbers of highly expressed genes (RPKM values > 1,000) and differentially expressed genes (|log_2_FC| > 1 and *p* < 0.05) in strains 60473 and OSK2A are presented. In addition, RPKM values for genes involved in the catabolic and EET pathways (Figure 3) are summarized in Table 1.

**Table 1:** A heat-map table showing RPKM and log_2_FD values for genes considered to be involved in current generation from acetate under the three growth conditions.^1^

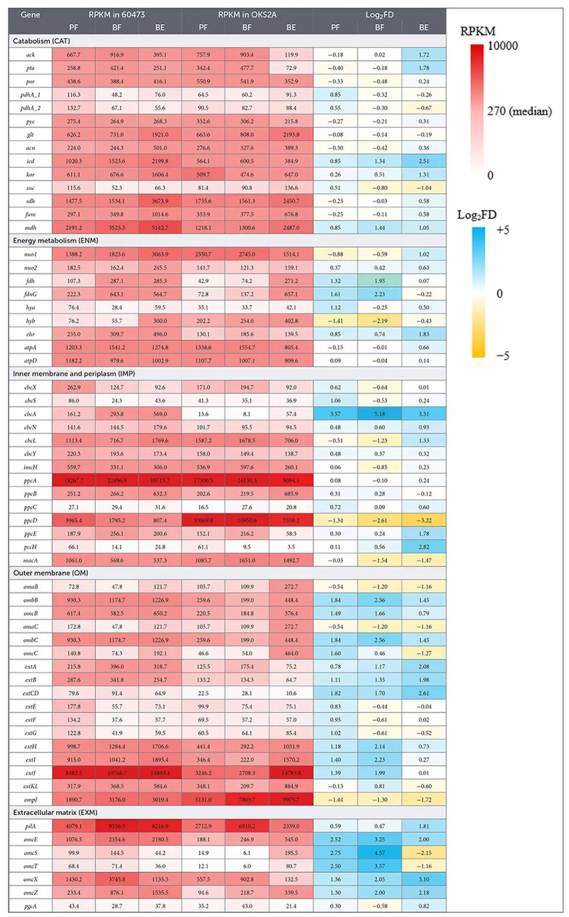

^1^Data are means of data obtained from three independent cultures.

Highly expressed genes in strains 60473 and OSK2A under the three growth conditions are listed in Supplementary Tables 5–10. These tables generally include genes for ATP synthase (*atp*), NADH:quinone oxidoreductase (*nuo1*) and some TCA-cycle enzymes, such as malate dehydrogenase (*mdh*) and succinate dehydrogenase (*sdh*). It is interesting that *mdh* and *sdh* are highly expressed even in the absence of fumarate (the BE condition). Among genes encoding putative components of the EET pathways, *cbcL*, *ppcA*, *ombB*, *extHIJ*, *omcE*, *omcZ*, and *pilA* were detected as highly expressed genes. Based on genes that are highly expressed under the BE condition (Table 1), a major route of electrons in the EET pathway of strain 60473 is predicted as follows: NADH → Nuo1 → quinones → CbcL → PpcA → ExtHIJKL and PilA/OmcE/OmcZ → electrode. Although *omcE* and *omcZ* were not listed up as highly expressed genes in strain OSK2A under the BE condition, a major route of electrons in OSK2A would be the same as that of 60473. Among them, CbcL is known to be an inner membrane cyt-c that is required for the reduction of low potential (e.g., −0.1 V vs. SHE) electron acceptors (Zacharoff et al., 2016). Since the present work measured current using WE poised at 0 V vs. SHE, it is reasonable to assume that these strains mainly utilized CbcL for EET toward WE. PpcA is one of periplasmic cyts-c that connects IM and OM electron conduits (Lloyd et al., 2003). A study has shown that *ppcA* is highly expressed in strain PCA (Choi et al., 2022), while gene-knockout examinations show that PpcABCDE cyts-c have redundant functions (Choi et al., 2022). Among five OM PCCs, strains 60473 and OSK2A seemed to express *extHIJKL* at the highest level, whereas it has been reported that strain PCA expresses *omaB/ombB/omcB* at the highest level with electrodes as electron acceptors (Jiménez Otero et al., 2018). On the other hand, gene-knockout tests have shown that ExtABCD is necessary for strain PCA to grow on extracellular electron acceptors (Jiménez Otero et al., 2018). It is suggested that how PCCs are utilized varies among *G. sulfurreducens* strains.

Regarding genes for EET components in the extracellular matrix (EXM), *pilA*, *omcE*, *omcX* and *omcZ* were highly expressed (Table 1). *pilA* encodes the major component of conductive pili that are the most abundant conductive filaments expressed by *G. sulfurreducens* (Liu et al., 2021) and necessary for long-range EET (Nevin et al., 2009; Vargas et al., 2013). *omcE* and *omcZ* are known to encode cyts-c that constitute conductive nanowires (Zakizadeh Tabari and Hochbaum, 2025). Among them, the OmcZ nanowire has been shown to be essential for optimal current production by *G. sulfurreducens* (Kai et al., 2021). On the other hand, OmcE has been shown to interact with resorufin, an artificial electron mediator, and promote EET (Qin et al., 2024), while it is unclear how OmcE contributes to current generation in the absence of electron mediators. A recent work on *G. sulfurreducens* PCA has suggested that periplasmic cyts-c PpcABCDE transfer electrons directly to OmcS nanowires in the periplasmic space (Portela et al., 2024), and it is likely that similar electron transfer occurs between PpcA and OmcE/OmcZ. Regarding *omcX*, a metatranscriptomic analysis of anaerobic sludge dechlorinating volatile chlorinated hydrocarbons detected abundant expression of this gene in *Geobacter* (Chen et al., 2019), while no information is available for its roles in EET.

RNA-seq also identifies genes that are differentially expressed in association with biofilm formation and/or current generation (Supplementary Tables 11–14). It should be stated that *omcZ* and *pilA* were up-regulated in association with biofilm formation in strains 60473 and OSK2A, while *omcX* and *omcE* were up-regulated in association with biofilm formation only in strains 60473. In association with current generation, some cyt-c genes were up-regulated; these include *omcM*, *omcC*, *omaC*, *cbcL, pccB*, *omcH*, *omcG*, *omcJ* and GEO60473_06650 for strain 60473, and *omcS*, *omcT*, *omcC*, *extI*, GSUET_07320, *extJ*, GSUET_30550, *extH*, *omcJ*, *ppcB*, GSUET_11710, *omcE* and some others for strain OSK2A. *omcC*, *pccB* and *omcJ* are up-regulated in both strains. Among them, it has been reported that an *omcJ* deletion mutant of PCA showed impaired growth on insoluble Fe(III) but not on soluble Fe(III) (Aklujkar et al., 2013). It is therefore likely that OmcJ would be involved in EET, while its function remains not to be identified. Attention should however be paid to the discussion on the involvement of differentially expressed genes in EET, since many of these were expressed at low levels.

### 3.5 Comparative transcriptomics of strains 60473 and OSK2A

**FIGURE 4:**
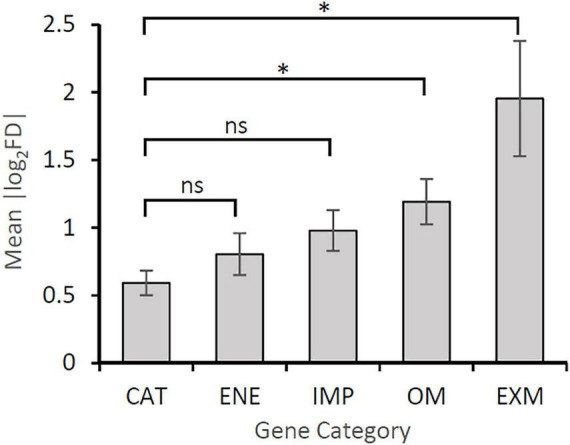
Comparison in average values for fold differences (FDs) between strains 60473 and OSK2A in the expression of genes (RPKMs) in different categories. Gene categories are as follows: CAT, genes for catabolism; ENM, genes for energy metabolism; IMP, genes for electron transfer across the inner membrane and periplasm; OM, genes for electron transfer across the outer membrane; EXM, genes for electron transfer in the extracellular matrix. Genes in each category are listed in Supplementary Table 3. An error bar represents SE. *, significant difference; ns, no significant difference.

In order to gain insights into molecular mechanisms behind the difference in current generated by strains 60473 and OSK2A, we also compared expression levels (RPKM values) of orthologous catabolic and EET genes between these strains using log_2_FD values as indices (Table 1). We aimed at identifying genes whose expression levels are largely different between these strains. It is seen in Table 1 that differences in expression levels (|log_2_FD|) for genes encoding EET proteins seem to be generally larger than those for catabolic enzymes. We therefore determined mean |log_2_FD| values of genes in each category in Table 1, and results are shown in Figure 4. The figure clearly shows that the mean |log_2_FD| values for the OM and EXM genes are significantly larger than that for the catabolic (CAT) genes, among which that for the EXM genes is particularly large. This result suggests that the way EXM components are utilized varies even among closely related strains of *G. sulfurreducens*, resulting in differences in EET activities (e.g., current densities).

**FIGURE 5:**
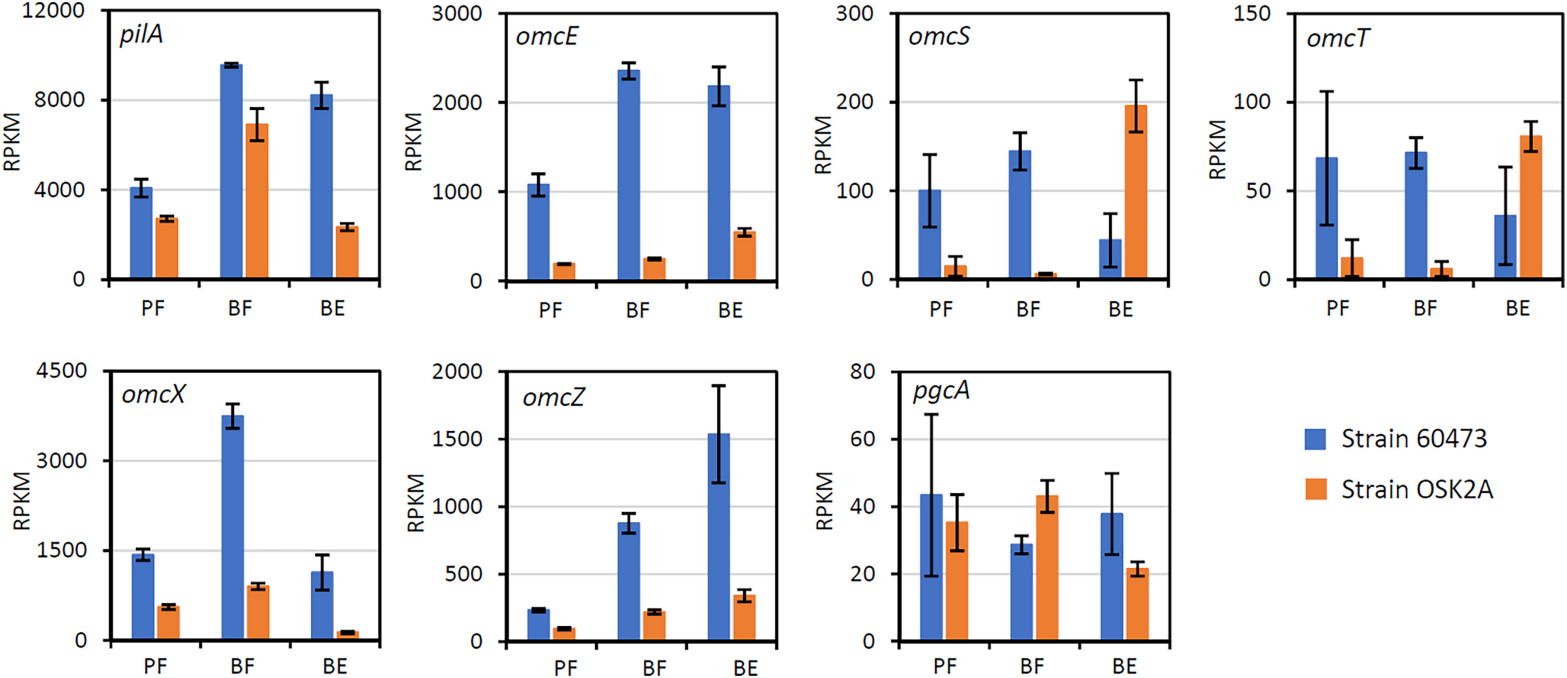
RPKM values showing expression levels of genes considered to be involved in electron transfer in the extracellular matrix of strains 60473 and OSK2A under the three growth conditions (PF, BF, and BE; refer to the methods section for abbreviations). An error bar represents SD (*n* = 3).

Comparisons in expression levels (RPKM values) of the EXM genes between strains 60473 and OSK2A under the three growth conditions are presented in Figure 5. It is shown that expression levels of *pilA*, *omcE*, *omcX*, and *omcZ* in strain 60473 under the BE condition are substantially higher than those in strain OSK2A. This result suggests a possibility that the high current density attained by strain 60473 is ascribable to the abundant expression of these genes. The *omcZ* gene was substantially up-regulated in association with current generation in strain 60473, suggesting its importance for current generation. On the other hand, *pilA* and *omcE* were up-regulated in association with biofilm formation, suggesting that PilA and OmcE not only contribute to current generation but also to biofilm formation. Although *omcS* and *omcT* were up-regulated in association with current generation in strain OSK2A, expression levels of these genes were low, suggesting their minor roles in current generation in these strains. From these results, we are interested in examining the idea that over production of PilA, OmcE and OmcZ would improve electrochemical activities of *G. sulfurreducens* strains.

Since expression levels of *pilA*, *omcE*, *omcX* and *omcZ* under the BE condition were largely different between strains 60473 and OSK2A, we compared nucleotide sequences of intergenic regions upstream of these genes between the two strains (Supplementary Figures 4A–D). We consider regulatory elements for these genes are present in these regions, since these are sufficiently long to include regulatory sequences, and neighboring genes are functionally unrelated. Surprisingly, regarding the *pilA*, *omcX*, and *omcZ* genes, the upstream sequences are 100% identical between the two strains, and only several nucleotide substitutions are present in the upstream region (approx. 500 bp) of *omcE*. We therefore consider that the large differences in the expression levels of these genes in strains 60473 and OSK2A are not attributable to nucleotide substitutions in regulatory sequences. It is also noteworthy that the genomes of these two strains encode highly conserved *pilS*/*pilR* (over 95% identical to those of strain PCA) whose products are regulators of *pilA* expression (Hernández-Eligio et al., 2017). In order to understand why expression levels of these genes are different between these strains, further studies are necessary for identifying sensing and regulatory mechanisms, such as riboswitches (Nelson et al., 2015), that control the transcription of these genes.

### 3.6 Implications

Bacteria have huge repertoire of metabolic activities, many of which are industrially useful. In order to understand physiological traits and molecular bases of industrially useful bacteria (IUB), microbiologists use representative strains of IUB (e.g., *G. sulfurreducens* strain PCA) as study models. On the other hand, it has also been known that even closely related strains of IUB in same genera and species, such as those affiliated with *G. sulfurreducens*, exhibit different levels of activities, while knowledge on molecular mechanisms behind strain-level variations in IUB is limited. Such knowledge would however facilitate the development of strategies for molecular breeding of IUB, leading to the development of high-performance industrial bioprocesses. We consider that strains affiliated with *G. sulfurreducens* would serve as models for studying molecular bases of strain-level variations in IUB, which would provide broad insights into applied microbiology.

The present work shows that expression levels of genes coding for extracellular proteins, including the OmcE and OmcZ nanowires, are largely different between the closely related *G. sulfurreducens* strains. Since these proteins are present only in *G. sulfurreducens* and related *Geobacter anodireducens*, it is likely that *G. sulfurreducens* has acquired these genes relatively recently during the course of evolution. Given the large variations in transcription levels of these genes, we assume that *G. sulfurreducens* strains have not yet undergone maturation in using these cyt-c nanowires, resulting in the variations in EET activities, such as current densities attained in BESs. Likewise, it is conceivable that strain-level variations in the expression of specifically distributed genes would occur in other bacteria, resulting in strain-level variations in phenotypes. It is therefore suggested that engineering of the expression of specifically distributed genes in IUB, such as genes for cyt-c nanowires in *G. sulfurreducens* (Wang et al., 2023), would be a possible strategy for constructing active IUB.

## Data Availability

The datasets presented in this study can be found in online repositories. The names of the repository/repositories and accession number(s) can be found in the article/Supplementary material.

## Footnotes

1
www.ncbi.nlm.nih.gov/datasets/genome/

2
https://www.genome.jp/tools-bin/clustalw
