# Supplementary material for: Comparative transcriptomics of closely related Geobacter sulfurreducens strains uncovers strain-level variations in extracellular electron transfer
Source: Front Microbiol. 2026 Apr 16;17:1795027. doi: 10.3389/fmicb.2026.1795027 (PMC13130476; doi:10.3389/fmicb.2026.1795027)
Supplement: Supplementary file 1 [file Data_Sheet_1.docx]

Supplementary Material

# Supplementary Figures

**Supplementary Figure S1.** Growth of strains 60473 and OSK2A. (A) Growth on acetate and fumarate as electron donor and acceptor, respectively. (B) Growth on acetate and ferric ion as electron donor and acceptor, respectively. Datum points are means of OD_600_ values for two or three independent cultures.


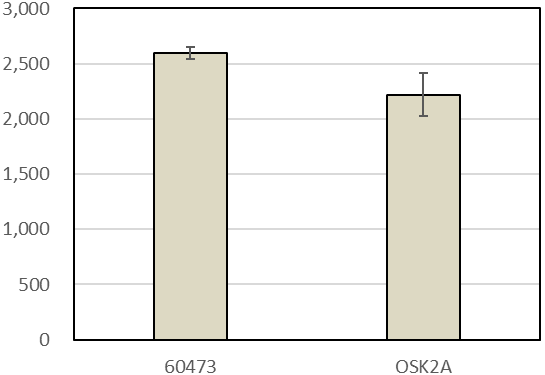


Protein (μg per WE)

**Supplementary Figure S2.** Amounts of proteins attaching onto WEs. Data are means of three ECs, and error bars represent SDs.

**60473**

**OSK2A**

**Supplementary Figure S3.** Comparative Synteny map showing homologies of genes in sequence and order between strains 60473 and OSK2A. Sequence homologies are presented with colors as shown above the map.

GEO60473_09100/GSUET_21310

60473 AGCTAACGTTCCGGTCTTTCCGTTATCGCTTGGCAAAATTCGGCATGGATGAGGAGTGAA

OSK2A AGCTAACGTTCCGGTCTTTCCGTTATCGCTTGGCAAAATTCGGCATGGATGAGGAGTGAA

************************************************************

60473 GAGGAGCCAGTGACGAAAATCGTCAGACACAAGTGACGAAATAGGTGGTGAAGTGGCAGG

OSK2A GAGGAGCCAGTGACGAAAATCGTCAGACACAAGTGACGAAATAGGTGGTGAAGTGGCAGG

************************************************************

60473 TTGAAGCGGTTGCGTTGTGTAAAACGCTGAAATTATAGCCATGTATAAGTTGGTTCGGCT

OSK2A TTGAAGCGGTTGCGTTGTGTAAAACGCTGAAATTATAGCCATGTATAAGTTGGTTCGGCT

************************************************************

60473 TTTGCTATGTTCACGATAACGTTTAAGGATTAAACGGATAATTGGCCAATTACCCCCATA

OSK2A TTTGCTATGTTCACGATAACGTTTAAGGATTAAACGGATAATTGGCCAATTACCCCCATA

************************************************************

GEO60473_09110/GSUET_21320

60473 CCCCAACACAAGCAGCAAAAAGAAGAAAGGAGACACTTATGCTTCAGAAACTCAGAAACA

OSK2A CCCCAACACAAGCAGCAAAAAGAAGAAAGGAGACACTTATGCTTCAGAAACTCAGAAACA

************************************************************

**Supplementary Figure S4.** (A) Alignment of nucleotide sequences in the intergenic regions upstream of *pilA* genes (GEO60473_09110 and GSUET_21320) in the genomes of strains 60473 and OSK2A, respectively. Coding sequences are indicated with blue letters. Putative ribosome-binding sites are indicated with underlines.

GEO60473_00740/GSUET_07630

60473 CATCTCCGACGATTTCCTCAAGCGCAACCCCATCCCGGGATGGCAGGAGTAATATCAGTT

OSK2A CATCTCCGACGATTTCCTCAAGCGCAACCCCATCCCGGGATGGCAGGAGTAATATCAGTT

************************************************************

60473 TCATCACCTGCCAGGAGTCTGTCGGACTTGGGAAATCGAAGCGAAAGCGCTGCTGGGCCA

OSK2A TCATCACCTGCCAGGAGTCTGTCGGACTTGGGAAATCGAAGCGAAAGCGCTGCTGGGCCA

************************************************************

60473 GGGCTGCTTGTGTCGACTTGTGCGGGTCAATAGTCATTTTATTGGCCGGAAACCCGGCGA

OSK2A GGGCTGCTTGTGTCGGCTTGTGCGGGTCAATAGTCATTTTATTGGCCGGAAACCCGGCGA

*************** ********************************************

60473 AATATGAACCGGCCAGACGGATTTGCAGCAGCTTTATGGTAAGTCCGGCAGACGCCTGGA

OSK2A AATATGAACCGGCCAGACGGATTTGCAGCAGTTTTATGGTAAGTCCGGCAGACGCCTGGA

******************************* ****************************

60473 ACCCGCGGTTTCCGAAGAGCCCTCGCCAGCATGGTTGCTGTCGGGGGCCTTCTTATTTTT

OSK2A ACCCGCGGTTTCCGAAGAGCCCTCGCCAGCATGGTTGCTGTCGGGGGCCTTCTTGTTTTT

****************************************************** *****

60473 GTCATCAATAAGCTATTTCGCGGATGCATTGAGTGCCGGTGCCATCTTCCGTCCGGTGTT

OSK2A GTCATCAATAAGCTATTTCGCGGATGCATTGAGTGCCGGTGCCATCTTCCGTCCGGTGTT

************************************************************

60473 GTAGCGAATTGCGGTTGGCGCCAAAAAAATGTAGCGCTATATGGCGTATGATCTTCTCGG

OSK2A GTAGCGAATTGCGGTTGGCGCCAAAAAAATGTAGCGTTATATGGCGTATGATCTTCTCGG

************************************ ***********************

60473 AAATACGTCCCGGGACAACGTTGCCGAGAGAAATGGGCTTGGTTGGTGTGATTCGTGTGC

OSK2A AAATACGTCCCGGGACAACGTTGCCGAGAGAAATGGGCTTGGTTGGTGAGATTCGTGTGC

************************************************ ***********

60473 GCGCAACTATCCGTAATTATTTATTTACAGGCCTTTTATGAGAATACGTTGAATATTGGC

OSK2A GCGCAACTATCCGTAATTATTTATTTACAGGTCTTTTATGAGAATACGTTGAATATTGGC

******************************* ****************************

60473 GTGCTGGTTGCATTGTGTTTCTTCATCCATTGGTGTCAACGCGAACGGCTGAGAAAGCAC

OSK2A GTGCTGGTTGCATTGTGTTTCTTCATCCATTGGTGTCAACGCGAACGGCTGAGAAAGCAC

************************************************************

GEO60473_00750/GSUET_07640

60473 ATAGTACTCACGTGAAAGGAGAACGCAGAATGAGAAGCGAAGTAAAAATCGGATTGGCCC

OSK2A ATAGTACTCACGTGAAAGGAGAACGCAGAATGAGAAGCGAAGTAAAAATCGGATTGGCCC

************************************************************

**Supplementary Figure S4.** (B) Alignment of nucleotide sequences in the intergenic regions upstream of *omcE* genes (GEO60473_00750 and GSUET_07640) in the genomes of strains 60473 and OSK2A, respectively. Coding sequences are indicated with blue letters. Putative ribosome-binding sites are indicated with underlines.

GE60473_01240/GSUET_08120

60473 ACTGAGCCGGGCACCCCCGCCCGTAACCCTTTTTCAATCAGAGAACACCGAGCATGTGAC

OSK2A ACTGAGCCGGGCACCCCCGCCCGTAACCCTTTTTCAATCAGAGAACACCGAGCATGTGAC

************************************************************

60473 CACAAAGTCCACTTCACGTTATTCCCTTGCAATTTCAACAAGTTCAGCCCCCACTGAATT

OSK2A CACAAAGTCCACTTCACGTTATTCCCTTGCAATTTCAACAAGTTCAGCCCCCACTGAATT

************************************************************

60473 ACTTTTTTCTAATTGACTGAACCCCCCAGAAATGGCATAATCTCCCCGCTTTTCTATCGG

OSK2A ACTTTTTTCTAATTGACTGAACCCCCCAGAAATGGCATAATCTCCCCGCTTTTCTATCGG

************************************************************

60473 TTTGCTCCGGGATATTCCCGCGACTCACGCTCTCTTCCGTCCGGAAGCGGAGCACATACT

OSK2A TTTGCTCCGGGATATTCCCGCGACTCACGCTCTCTTCCGTCCGGAAGCGGAGCACATACT

************************************************************

GE60473_01250/GSUET_08130

60473 ATTCGTGCAAAACGCACAAAGGAGGAACGATGAACGCTGTACGACGTATCAGGCTCGTTG

OSK2A ATTCGTGCAAAACGCACAAAGGAGGAACGATGAACGCTGTACGACGTATCAGGCTCGTTG

************************************************************

**Supplementary Figure S4.** (C) Alignment of nucleotide sequences in the intergenic regions upstream of *omcX* genes (GEO60473_01250 and GSUET_08130) in the genomes of strains 60473 and OSK2A, respectively. Coding sequences are indicated with blue letters. Putative ribosome-binding sites are indicated with underlines.

GEO60473_15220/GSUET_15430

60473 ATATCAATGGCGATTCCCGTGTGATCGTTGCAGATAGGGCATTTCATGGCTCTTTTCTCCTTTGAGTTGCTTTCTTTTACTAAACTATAG

OSK2A ATATCAATGGCGATTCCCGTGTGATCGTTGCAGATAGGGCATTTCATGGCTCTTTTCTCCTTTGAGTTGCTTTCTTTTACTAAACTATAG

******************************************************************************************

60473 AAGAGATTAGATGGGCAAACAAGAGGTAAAATACATATAAATATCACTAAAACAATAGTTTACGCCTGCAGGTGGCCTCGAAGCGTACAC

OSK2A AAGAGATTAGATGGGCAAACAAGAGGTAAAATACATATAAATATCACTAAAACAATAGTTTACGCCTGCAGGTGGCCTCGAAGCGTACAC

******************************************************************************************

60473 CGCCATTATGCTGTCTCGCTCGAACGACTAACAGCGAGGATACGAAGGGTAGTCCTAATACTCTGAATTTACGGGCGAATCGTGGATAAA

OSK2A CGCCATTATGCTGTCTCGCTCGAACGACTAACAGCGAGGATACGAAGGGTAGTCCTAATACTCTGAATTTACGGGCGAATCGTGGATAAA

******************************************************************************************

60473 ACGAAATTATGTATACATGATCGGTAGTTGGACTGATTTTCGAGAATCGGGCAGCTGAGATCGCAAAAAAAATATCAGGTGCAATTTTCA

OSK2A ACGAAATTATGTATACATGATCGGTAGTTGGACTGATTTTCGAGAATCGGGCAGCTGAGATCGCAAAAAAAATATCAGGTGCAATTTTCA

******************************************************************************************

60473 AACTGAAGTTAGGGAAATTGCGCATGAGATTCGGACTGACCCTGTTGTGACTCATCAAATCGCCTGGGATGACTCCATATGCCTTCCATT

OSK2A AACTGAAGTTAGGGAAATTGCGCATGAGATTCGGACTGACCCTGTTGTGACTCATCAAATCGCCTGGGATGACTCCATATGCCTTCCATT

******************************************************************************************

60473 GGATCAAAAATTTCGGCTCGACGCTTGCAATTTTGAGATAGTGTTAACATTAGAGGATATGGTTGTTGGCAGTAATTGAATATATTAATT

OSK2A GGATCAAAAATTTCGGCTCGACGCTTGCAATTTTGAGATAGTGTTAACATTAGAGGATATGGTTGTTGGCAGTAATTGAATATATTAATT

******************************************************************************************

60473 AAATGCAACATGCTGTGTTTTCGTTGTTTCATGTAAGTTTGGTAATGATTCAGTCGCAAGTGCCGCCTTTAAGGGGCTGGCTATTTCACG

OSK2A AAATGCAACATGCTGTGTTTTCGTTGTTTCATGTAAGTTTGGTAATGATTCAGTCGCAAGTGCCGCCTTTAAGGGGCTGGCTATTTCACG

******************************************************************************************

60473 GTGACGGCAGTCAATTGACGTAAAATTGACCGGCTTGATCCGGTCAGATAGGTGCTGCTAGTCTGTCGCTGTTAATTATGTGTAGTAAAA

OSK2A GTGACGGCAGTCAATTGACGTAAAATTGACCGGCTTGATCCGGTCAGATAGGTGCTGCTAGTCTGTCGCTGTTAATTATGTGTAGTAAAA

******************************************************************************************

60473 TAGCGTATCAGGCGAGCTTGGTGGAAGAGAACGTCAAATTCTTGTCTGGCGGGATTGCAGGTATGTGTGTTGCTAATTTGTTGCTGAACG

OSK2A TAGCGTATCAGGCGAGCTTGGTGGAAGAGAACGTCAAATTCTTGTCTGGCGGGATTGCAGGTATGTGTGTTGCTAATTTGTTGCTGAACG

******************************************************************************************

60473 TTAAAATGGCCTTTAAAAGTATGTTGACAAAAAGCTCTGAATAGCTACAGTACACAGTTGTGTTTATCCTGCTTAATGATATATGTGATG

OSK2A TTAAAATGGCCTTTAAAAGTATGTTGACAAAAAGCTCTGAATAGCTACAGTACACAGTTGTGTTTATCCTGCTTAATGATATATGTGATG

******************************************************************************************

60473 TGATATCCCGGCCCTGCTCGCCCCCTGAACACAACAAGGTACCATCTGAAAAATCTGCCCGAAGACCATTCCGGCCTGCGCATCGCCGGA

OSK2A TGATATCCCGGCCCTGCTCGCCCCCTGAACACAACAAGGTACCATCTGAAAAATCTGCCCGAAGACCATTCCGGCCTGCGCATCGCCGGA

******************************************************************************************

60473 CGCAGAGACATGCGGGTCTGCCGGTCAATGTGTATGCGTCCTGTTGCGGTAGTCCGATGCAGCGCCTTTTAGTCGCAGAACCATGCATTG

OSK2A CGCAGAGACATGCGGGTCTGCCGGTCAATGTGTATGCGTCCTGTTGCGGTAGTCCGATGCAGCGCCTTTTAGTCGCAGAACCATGCATTG

******************************************************************************************

60473 AATTTGCCAGAGTGAGGGGACATGACATGCCGAAAGGAGGTGGCAAGGAGGAAGGAATACGTCAATCGCTTTGAATGTAATGTTAATCTT

OSK2A AATTTGCCAGAGTGAGGGGACATGACATGCCGAAAGGAGGTGGCAAGGAGGAAGGAATACGTCAATCGCTTTGAATGTAATGTTAATCTT

******************************************************************************************

GEO60473_15210/GSUET_15420

60473 TCATAATGGAGTCTTCACAAGAAAGGAGCAGAAAGGAATGAAGAAAAAGGTACTGATTGGCGCATCGCTCGCAGCCGTTGTCTTAACCGG

OSK2A TCATAATGGAGTCTTCACAAGAAAGGAGCAGAAAGGAATGAAGAAAAAGGTACTGATTGGCGCATCGCTCGCAGCCGTTGTCTTAACCGG

******************************************************************************************

**Supplementary Figure S4.** (D) Alignment of nucleotide sequences in the intergenic regions upstream of *omcZ* genes (GEO60473_15210 and GSUET_15420) in the genomes of strains 60473 and OSK2A, respectively. Coding sequences are indicated with blue letters. Putative ribosome-binding sites are indicated with underlines.
